# Glycolic acid-based deep eutectic solvents boosting co-production of xylo-oligomers and fermentable sugars from corncob and the related kinetic mechanism

**DOI:** 10.1186/s13068-023-02369-1

**Published:** 2023-08-07

**Authors:** Hai-Qing Deng, Xiao-Hui Lin, Jun-Tao Fan, Ping-Zhang Fu, Jia-Jun Guan, Han-Lin Lei, Li-Hao Liu, Lin-Hao Lai, Xue-Dan Hou, Wen-Yong Lou

**Affiliations:** 1 School of Biomedical and Pharmaceutical Sciences, Guangdong University of Technology, Guangzhou, 510006 China; 2 Guangdong Provincial Key Laboratory of Plant Resources Biorefinery, Guangdong University of Technology, Guangzhou, 510006 China; 3 Lab of Applied Biocatalysis, School of Food Science and Engineering, South China University of Technology, Guangzhou, 510640 Guangdong China

**Keywords:** Corncob, Deep eutectic solvents, Xylo-oligomers, Metal ions, Kinetic mechanism

## Abstract

**Background:**

Xylo-oligomers are a kind of high value-added products in biomass fractionation. Although there are several chemical methods to obtain xylo-oligomers from biomass, the reports about the deep eutectic solvents (DESs)-mediated co-production of xylo-oligomers and fermentable sugars and the related kinetic mechanism are limited.

**Results:**

In this work, glycolic acid-based DESs were used to obtain xylo-oligomers from corncob. The highest xylo-oligomers yield of 65.9% was achieved at 120 °C for 20 min, of which the functional xylo-oligosaccharides (XOSs, DP 2–5) accounted for up to 31.8%. Meanwhile, the enzymatic digestion of cellulose and xylan in residues reached 81.0% and 95.5%, respectively. Moreover, the addition of metal inorganic salts significantly accelerated the hydrolysis of xylan and even the degradation of xylo-oligomers in DES, thus resulting in higher selectivity of xylan removal. AlCl_3_ showed the strongest synergistic effect with DES on accelerating the processes, while FeCl_2_ is best one for xylo-oligomers accumulation, affording the highest xylo-oligomers yield of 66.1% for only 10 min. Furthermore, the kinetic study indicates that the ‘potential hydrolysis degree’ model could well describe the xylan hydrolysis processes and glycolic acid/lactic acid (3:1) is a promising solvent for xylo-oligomers production, in particular, it worked well with FeCl_2_ for the excellent accumulation of xylo-oligomers.

**Conclusions:**

Glycolic acid-based deep eutectic solvents can be successfully applied in corncob fractionation with excellent xylo-oligomers and fermentable sugars yields on mild conditions, and the large amount of xylo-oligosaccharides accumulation could be achieved by specific process controlling. The strategies established here can be useful for developing high-valued products from biomass.

## Background

As one of the main agricultural wastes, corncob is produced in large quantities. It is considered to be an important raw material for the production of high-valued biochemicals and energy fuels. Like the common lignocellulosic biomass, corncob is mainly composed of three major components, namely, cellulose, hemicellulose, and lignin [1, 2]. Hemicellulose of biomass is more sensitive and easily to be hydrolyzed; however, it is often neglected, because it is difficult to obtain high value-added products from it [3]. Hemicellulose is a miscellaneous polysaccharides containing branched polymers. It contains three types, and 65–85% of hemicellulose is made up of xylan backbones [4].The hydrolysis products of xylan include low xylan fragments (DP > 6), xylo-oligosaccharides (XOSs, DP < 6), and xylose, and the health benefits of XOSs have been reporte, including lowering blood cholesterol, increasing calcium absorption, antioxidant effects, maintaining gastrointestinal health, and reducing the risk of colon cancer. Also, they have toxic effects on human leukemia cells, and are benefits for patients suffering from type 2 diabetes [5]. Currently, there are different ways to obtain XOSs from biomass. For example, acquiring XOSs from poplar [6–8] or corncob [9, 10] using acetic acid. Hydrothermal pretreatment or it related hybrid treatments were also employed to produce XOSs from biomass [11–13]. Besides, XOSs have also been extracted using aqueous solutions of metal inorganic salts, for instance, using FeCl_2_ and MgCl_2_ to co-catalyze xylan to obtain XOSs [14, 15]. DESs have been emerging as effective solvents for biomass deconstruction, pretreatment and conversion, because they are easy to be prepared, tunable, and environmentally friendly [16]. However, lots of DES-related biomass deconstruction mostly targeted toward fermentable sugars or lignin, and only limited reports focused on XOSs production. Except for the reported work in which DES was combined with lactic acid for the co-production of oligosaccharides [17]. Besides, lactic acid/choline chloride and hot water were reported to act on poplar synergistically for XOSs production with the highest yield of 66.5% [18]. Hence, DES-mediated XOSs production requires more concerns for a better understanding of the mechanism of XOSs accumulation in different DESs.

**Scheme 1:**
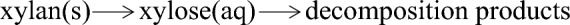
The Seaman’s model for xylan hydrolysis

**Scheme 2:**
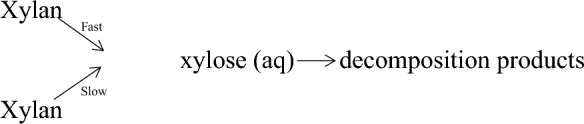
The two-step hydrolysis model for xylan hydrolysis

**Scheme 3:**
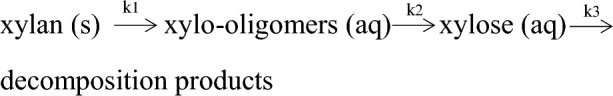
The ‘potential degree of hydrolysis (Hd)’ model for xylan hydrolysis

Kinetic study is helpful for exploring the process mechanism of XOSs accumulation. At present, many kinetic models of xylan dissolution have been reported in dilute acid hydrolysis system, which has been considered to be a homogeneous and irreversible first-order reaction [16]. Saeman’s model, the simplest model used widely, is often employed to fit the process of cellulose and xylan hydrolysis (Scheme 1) [19, 20]. While the two-step hydrolysis model believes that xylan hydrolysis can be divided into two parts, which including the fast-reacting part and the slow-reacting part, and the two parts proceed at the same time (Scheme 2) [21]. The two-step hydrolysis model has been able to describe the hydrolysis kinetic of xylan in lignocellulose, hardwoods, such as birch, poplar, maple, and aspen hardwoods, and grassy plants, such as bagasse, corn stover, and reed [20]. Because of the various distribution of xylan in lignocellulose and the different degrees of binding with cellulose and lignin, the difference of the xylan hydrolysis exists. The concept of ‘potential degree of hydrolysis (H_d_)’ is considered to be more accurate to describe the hydrolysis of xylan and the production of XOSs, and it also has been widely used (Scheme 3). For example, this model was successfully applied in xylan hydrolysis during dilute sulfuric acid treatment of bagasse to describe XOSs production and xylose degradation [16]. Besides, the solubilization of bagasse lignin and xylan in formic acid also can be fitted by the model [20]. However, there is currently no report about the kinetics of xylan hydrolysis and XOSs production during DES pretreatment.

In this work, glycolic acid-based novel DESs were explored to co-product XOSs and fermentable sugars from corncob. Factors, such as DES type, the molar ratio of glycolic acid to hydrogen-bonding acceptors (HBAs), metal inorganic salts, and treating time, were investigated. Then, kinetic study was conducted to explore the mechanism of xylan hydrolysis and XOSs accumulation in these DESs.

## Result and discussion

### Effect of DES on the composition and saccharification of corncob

The structure and property of DES have proven to be closely related to the deconstruction degree of biomass [22], especially for the acidic DES. The acidity of them correlated well with the hemicellulose or lignin removal [23]. In this study, some glycolic acid- based DES were used for corncob deconstruction to obtain fermentable sugars and XOSs. The results are shown in Table 1. Without addition of metal inorganic salts, the recovery rate of corncob residues varied in the range of 46.2–72.6% when the molar ratios of glycolic acid to HBAs were set at 1:1. The lignin and xylan removal ability follows the trend of GG > GL > GC, which is opposite to the residue recovery rates, and lignin and xylan contents, but in line with cellulose contents in the residues. Likewise, the enzymatic digestibility of the residues showed the same trend: GG > GL > GC, which is in accord with the trend of lignin and xylan removal rates. The more removal of lignin and xylan, the higher digestibility of the residues. GG (1:1) pretreatment of corncob at 120 °C for 20 min achieved the highest digestibility of 93.2% and 77.8% for cellulose and xylan, respectively. The strong biomass deconstruction ability of GG was probably attributed to the strong acidity and hydrogen-bonding accepting ability of guanidine hydrochloride.

**Table 1:** The composition and enzymatic hydrolysis of corncob residues treated by DESs

DES	Residues’ recovery (%)^a^	Composition of residues (%)^b^					Enzymatic hydrolysis of residues^c^	
		Cellulose	Xylan	AIL	ASL	Ash	Digestibility (%)	
							Cellulose xylan	
Raw	**–**	31.3	23.8	18.1	2.1	1.7	45.2	23.0
GC(1:1)	72.6	42.4	23.2	12.8	1.5	2.4	61.0	39.9
GL(1:1)	71.4	43.2	20.9	12.5	1.5	2.8	81.1	69.2
GG (1:1)	46.2	58.2	11.1	16.9	0.8	2.6	93.2	77.8
GC(3:1)	48.5	58.1	12.7	18.7	1.1	0.4	84.6	82.5
GL(3:1)	55.2	51.3	17.6	18.0	1.3	1.3	80.5	71.5
GG(3:1)	45.1	64.5	8.7	10.9	0.7	1.6	81.0	95.5
GC(3:1) + FeCl_2_.4H_2_O	52.6	55.8	14.2	15.8	1.3	1.1	82.3	78.0
GL(3;1) + FeCl_2_.4H_2_O	45.6	63.1	9.7	10.0	0.7	1.2	72.9	90.1
GG(3:1) + FeCl_2_.4H_2_O	41.0	63.1	9.7	12.6	0.8	0.8	82.2	92.3
GC(3:1) + AlCl_3_.6H_2_O	47.7	56.2	2.2	26.1	0.4	1.3	58.2	65.1
GL(3:1) + AlCl_3_.6H_2_O	37.3	67.2	3.3	21.7	0.3	1.0	35.1	42.8
GG(3:1) + AlCl_3_.6H_2_O	41.5	59.6	1.8	21.6	0.5	0.6	43.0	41.3
GL(3:1) + MgCl_2_.6H_2_O	45.0	64.3	9.7	10.8	0.6	1.2	72.1	81.9

*AIL* acid-insoluble lignin, *ASL* acid-soluble lignin

^a^Corncob was mixed with DESs in a 5wt% loading at 120 °C for 20 min, and then, the residues were washed by ethanol and water and freeze-dried before use. The data were present as mean value

^b^Determined via the NREL protocol (Sluiter et al. [38]). The results are expressed as a percentage of the residues. All data were showed as mean values with standard deviation less than 5%

^c^20 mg of recovered corncob, 7 mL of citrate buffer (50 mmol L^−1^, pH 4.8), and 8.3 U mL^−1^ cellulase from *Trichoderma reesei*; 50 °C and 120 rpm. The polysaccharides’ digestibilities were calculated on the results of 21 h hydrolysis. The data were presented as mean value

With the increase of the molar ratio of glycolic acid and HBAs to 3:1, the recovery rates of corncob residues were decreased to 45.1–55.2%. Obviously, more xylan and lignin were removed during the process, reflected by the lowered contents of xylan and lignin, especially for xylan (8.7–17.6% vs 11.1–23.2%), and also the increased cellulose contents (51.3–64.5% vs 42.4–58.2%). Similar results were observed in the previous studies, in which the removal rates of xylan increased with the increase of the acid molar ratios of DES [24]. It was also found that GG treatment led to the largest amount of xylan and lignin removal, which were 86.1% and 74.1%, respectively. However, the cellulose digestion decreased from 93.2 to 81.0% when the glycolic acid content in DES increased. This is possibly due to the formation of pseudo-lignin structures at harsher condition, which may be harmful for enzymes. It is worth to note that the digestibility of xylan in the residue significantly increased with the increase of the molar ratio of glycolic acid due to the large quantity of xylan removal. The best xylan digestion (95.5%) was also found in the case of GG (3:1). Interestingly, the digestibility of cellulose and xylan was 80.3% and 79.3%, respectively, when lactic acid:guanidine hydrochloride (1:1) used to treat rice straw at 120 °C for 6 h in our previous work [23]. In this work, with the same HBAs (guanidine hydrochloride), the digestibility of cellulose and xylan was comparable (81.1% and 69.2%, respectively), although the duration was shortened to 20 min, which greatly reduced the energy consumption.

As incorporating the metal inorganic salts into the above-mentioned DES (molar ratio was 3:1), the biomass deconstruction efficiency seems enhanced significantly. The recovery rates of the residues (41.0–52.6%) were declined when FeCl_2_ was added, and the xylan and lignin contents in the residues decreased accordingly due to the enhanced xylan and lignin removal ability of the new solvents system, except for the GC (3:1) case. Notably, when FeCl_2_ was replaced by AlCl_3_, the solvents showed highly selective power to remove xylan, because it was found that xylan contents were sharply decreased to the range of 1.8–3.3%, while the lignin contents increased and the recovery rate of residues lowered to 37.3–47.7% due to the larger amounts of xylan removal (83.3–95.6% vs 68.6–83.3%). When MgCl_2_ was included in DES, only GL (3:1) could form homogeneous solvent system, the deconstruction ability of this solvent is similar with GL (3;1)/FeCl_2_ for the comparable lignin, xylan removal rates, and enzymatic digestion efficiency of polysaccharides. In addition, the presence of FeCl_2_ led to slightly improvement in xylan and lignin removal, thus affording similar cellulose contents in the residues and then comparable polysaccharides’ digestibilities as compared to original ones. While AlCl_3_ appeared to be able to improve the interaction of HBAs or hydrogen-bonding donors (HBDs) with the key bonds between lignin and xylan, and especially accelerate the hydrolysis of xylan and even formation of furfural or over-degraded products like humins or pseudo-lignin, which may inhibit the cellulase activity, and thus, the apparently lowered digestibilities of cellulose and xylan were observed. This phenomenon was also reported in the literature, where the addition of AlCl_3_ to ethylene glycol resulted in a significant decrease of residue recovery and large amount of the degradation of hemicellulose and lignin in rice straw [25]. Simultaneously, the catalytic action of AlCl_3_ led to a high furfural yield [26]. Inorganic salts or metal chlorides, such as MgCl_2_, AlCl_3_, CaCl_2_, FeCl_3_, ZnCl_2_, and CuCl_2_, have been considered as excellent catalysts that could selectively catalytic and dissolve hemicellulose due to their Lewis acid character that can dissociate into complex ions [15, 27]. The metal ions may coordinate with water molecules to the glycosidic oxygen to help break down the glycosidic linkage, resulting in the reduction of reaction energy. Then, the coordinated water molecules of the metal cation complex acted as nucleophiles to generate hydrolysis products. In particular, trivalent salt ions like Al^3+^ were supposed to generate six-coordinate covalent bonds with water molecules, whereas divalent salts, such as Mg^2+^ and Fe^2+^ ions, can reach a steady complex ion by coordinating as a tetradentate ligand with an incomplete coordination [28]. Consequently, Al^3+^ system may produce more nucleophiles (coordinated water molecules) to promote the hydrolysis of glycosidic bonds than Fe^2+^ and Mg^2+^. It was also reported that FeCl_2_ as divalent inorganic salt has weaker catalytic activity on hemicellulose degradation than trivalent salts, and the latter could convert almost 100% xylan to xylose or furfural at 140–200 °C [29]. That is well agreed with the phenomenon that AlCl_3_ showed the strongest power toward xylan removal in the study.

### FT-IR characterization of the residues

**Fig. 1:**
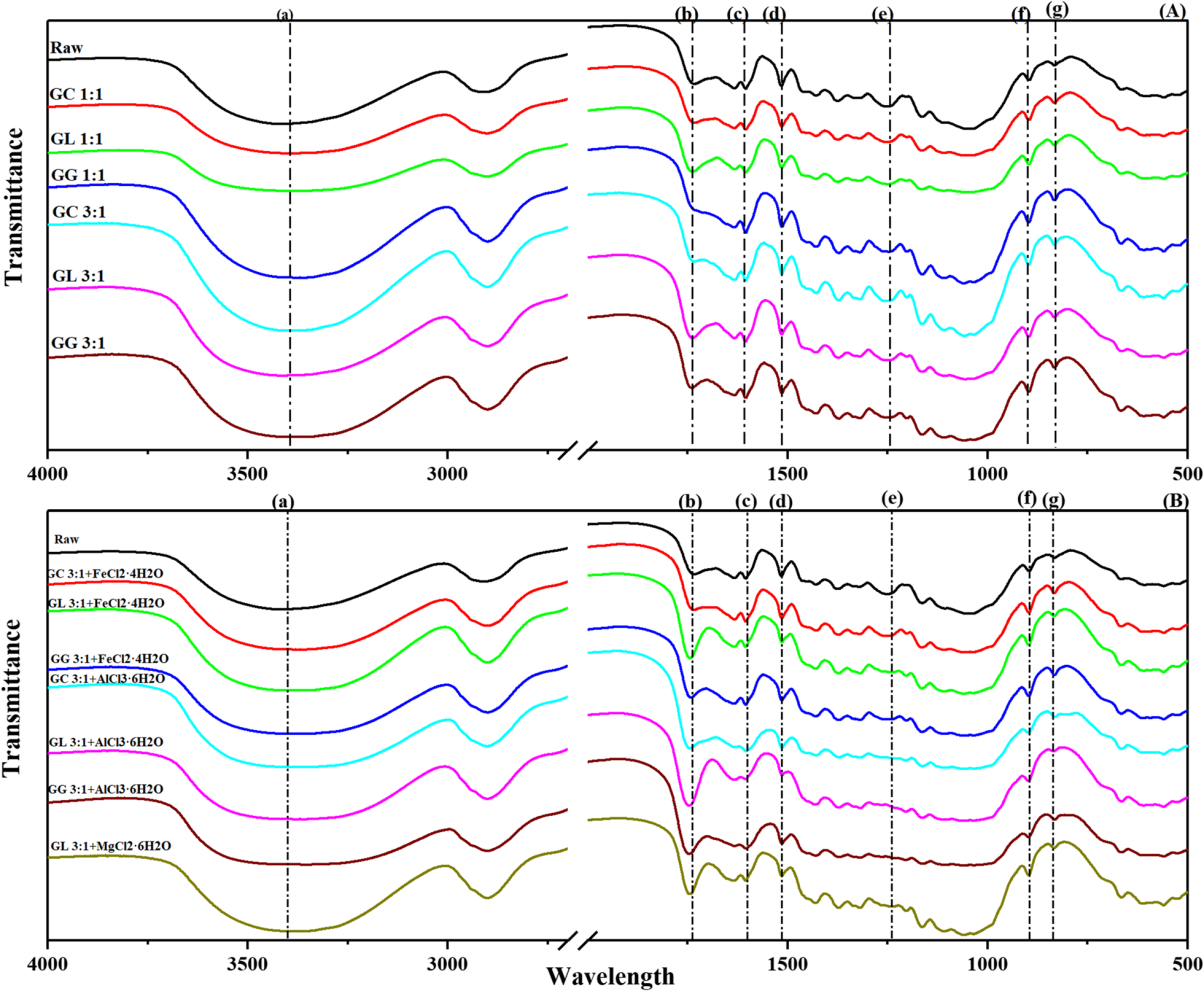
FT-IR spectra of corncob residues obtained from different DES treatment. **A** The basic DES systems; **B**. the metal inorganic salts incorporated DES systems. **a** 3387 cm^−1^; **b** 1732 cm^−1^; **c** 1608 cm^−1^; **d** 1512 cm^−1^; **e** 1249 cm^−1^; **f** 896 cm^−1^; **g** 834 cm^−1^

FT-IR spectra of residues obtained from different solvents’ treatment provide the information on the chemical structure changes. As shown in Fig. 1, the peaks at 3387 cm^−1^ are attributed to O-H-stretching vibration [30], the signals become broader and stronger, indicating the increase of cellulose content in the residues treated by DESs especially for those with higher molar ratio of glycolic acid. The peaks at 1608 cm^−1^ are assigned to lignin aromatic skeletal vibrations plus a C=O stretch, and the peaks at 1513 cm^−1^ and 834 cm^−1^ are from lignin aromatic skeletal vibrations and C–H out-of-plane vibrations in lignin, respectively [31]. Obviously, the intensity of these peaks only showed a slightly decline, which means that all the tested solvents could not remove large amounts of lignin for a short time treatment at 120 °C, which are consistent with the data in Table 1 (high preserved lignin in the residues). Also, the peaks at 1732 cm^−1^ are assigned to the ester linkages between lignin and carbohydrates [32] and these peaks weakened for the basic DES-treated residues especially for the GG (1:1) and GC (3:1), and further increase the pretreatment severity like using more acidic DES resulted in more preservation of ester linkages, which illustrates that moderate pretreatment severity is critical to cleave the ester linkages between lignin and carbohydrates and thus facilitating good enzymatic digestion of residues. Interestingly, the presence of metal inorganic salts resulted in the increased intensity of ester linkages of the residues. One possible explanation is that the inorganic salts-based DES treatment caused more xylan removal without extensively destroying the ester linkages between the lignin and hemicellulose. However, another likely reason is that some new esters formed between the degraded xylan or lignin fragments catalyzed by the metal inorganic salts [33]. In addition, the apparent breakage of linkages between lignin and hemicellulose after metal inorganic salts-based DES treatment was further confirmed by the reduced peak intensity at 1249 cm^−1^ (C-O-stretching signals in lignin and hemicellulose) [34], indicating that more xylan removal and some linkages between xylan and lignin were destructed probably caused by the synergistic effect of metal inorganic salts and DES. The signal at 898 cm^−1^ could be attributed to the *β*-glycosidic linkages between the sugar units in cellulose and hemicellulose [35], and the increase of the intensity of the peaks demonstrates the increased contents of polysaccharides in the recovered residues for all the tested DES cases. The results reveal that the removal of xylan and the preservation of most of the lignin occurred in the tested cases without significant chemical structure change of cellulose, and the synergistic catalysis effect of metal inorganic salts and DESs could enhance xylan removal and improve the DES dissolving selectivity toward biomass components.

### The xylo-oligomers production

**Fig. 2:**
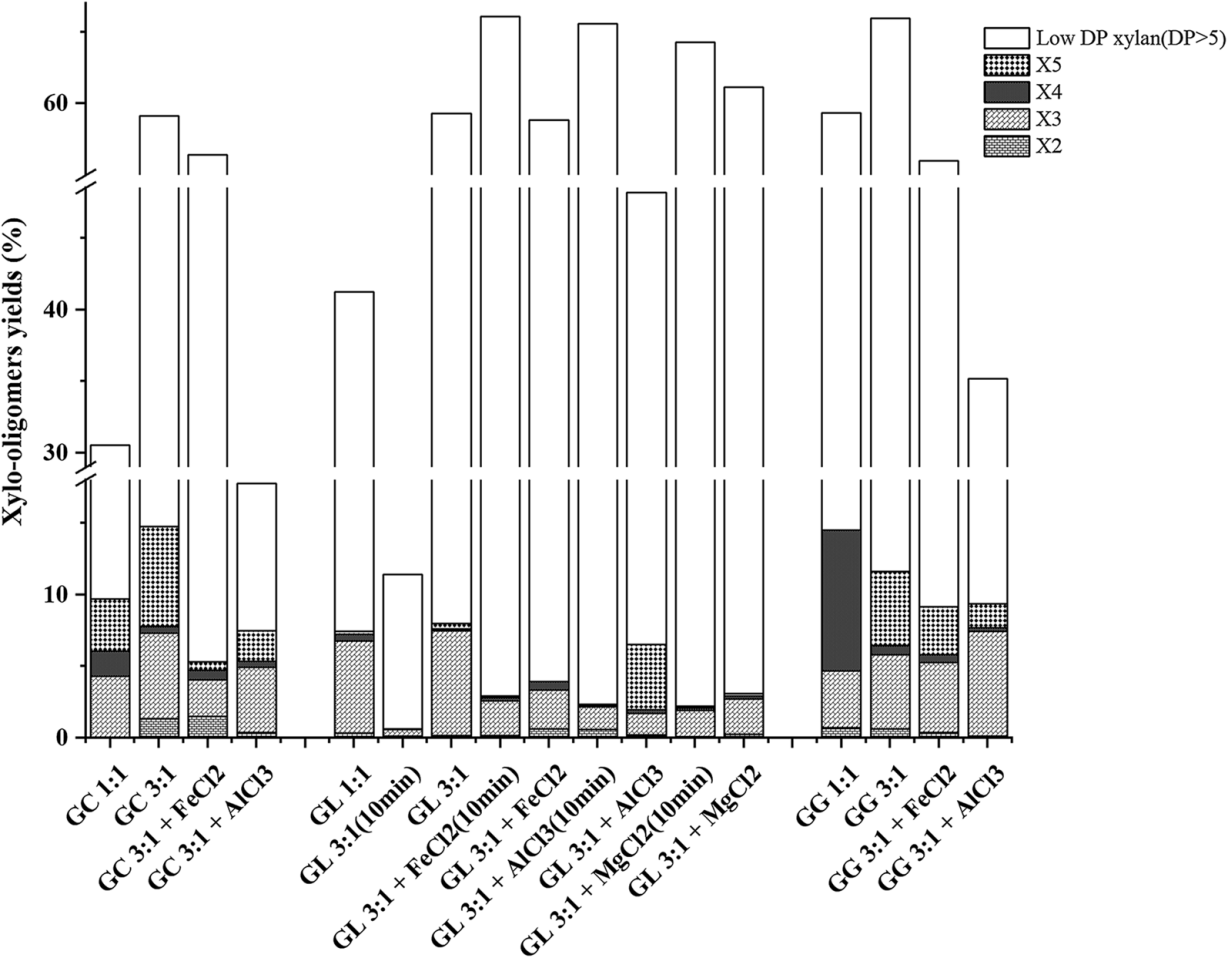
Production of xylo-oligomers from corncob treated with different solvent systems

**Fig. 3:**
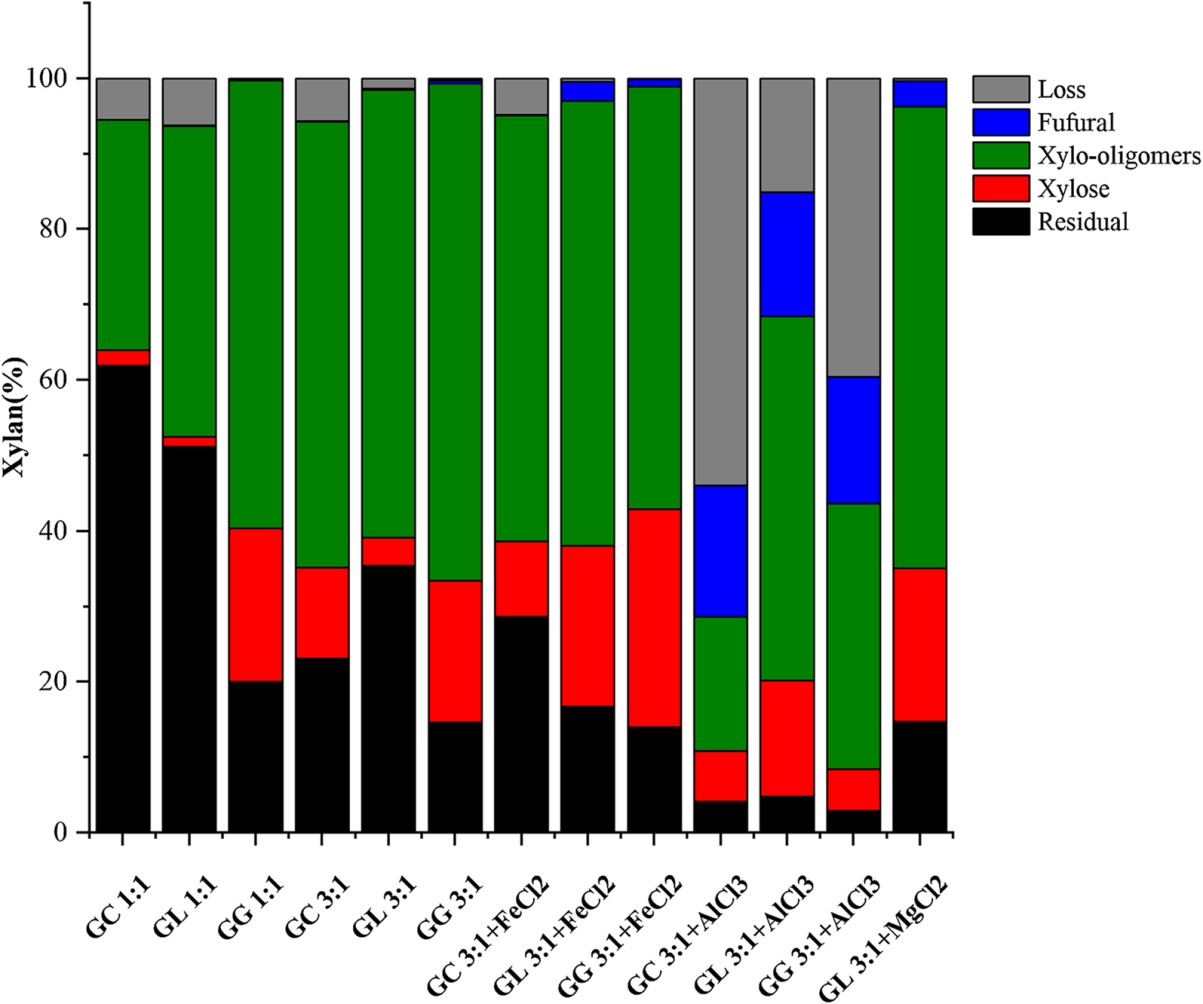
The mass balance of xylan

To date, there are various ways to obtain xylo-oligomers. For example, DESs were employed to obtain XOSs from poplar [18]; FeCl_2_ and MgCl_2_ were used to synergistically act on bagasse to extract XOSs [15]. To obtain XOSs, the total xylo-oligomers and XOSs (DP 2–5) in the pretreatment liquor were determined in this work, and the results are showed in Fig. 2. When the molar ratio of glycolic acid to HBAs was set at 1:1, the yield of total xylo-oligomers was varied in the range of 30.5–59.4%, which was significantly larger than the 26.0% of which obtained using lactic acid:choline chloride (2:1) to treat poplar at 130 °C for 90 min [18]. Besides, the total xylo-oligomers yields follow the trend of GG > GL > GC, which is  consistent with the order of biomass deconstruction ability of the solvents discussed before. Also, GG is the fiercest one for xylose releasing showed in Fig. 3. With the increase of the molar ratio (glycolic acid to HBAs) to 3:1, the yield of the total xylo-oligomers increased to the range of 59.1–65.9%, and the value for the solvent with poorer deconstruction capacity increased more significantly. GG (3:1) pretreatment achieved the highest total xylo-oligomers yield of 65.9% at 120 °C for only 20 min. With respect to the functional XOSs (DP 2–5) in the three types of DES-treated corncob hydrolysate, they account for 13.5–31.8% of the total xylo-oligomers in the liquor, and 7.5%-14.8% of xylan in raw biomass. GC is the best solvent to accumulate functional XOSs (DP 2–5), possibly due to the mildest condition of this system which can keep XOSs (DP 2–5) from over-degrading to xylose or humins. Specifically, the predominated fragments in GC, GL, and GG were X3 + X5, X3, and X3 + X5, respectively. It was found that GC treatment led to the formation of lots of larger fragments of xylo-oligomers, while the stronger acidic GL and GG caused more releasing of XOSs (DP < 6). Besides, it was obvious that increasing the molar ratio of glycolic acid to HBAs led to significant improvements in total xylo-oligomers yields for all the DESs, especially for GC (nearly two times of increase).

When metal inorganic salts were added into the basic DES (3:1 of the molar ratio of glycolic acid to HBAs), 20 min treatment led to the decrease of the total xylo-oligomers yield in the liquor except for the GL (3:1)/FeCl_2_.4H_2_O and GL (3:1)/MgCl_2_.6H_2_O-associated cases. The presence of metal ions especially for Al^3+^ caused the tremendous decline in the yield of the total xylo-oligomers and functional XOSs (DP 2–5) (Fig. 2), and simultaneously the apparent increase of xylose and even furfural production (Fig. 3). For instance, the total xylo-oligomers yield sharply decreased from 59.1% to 17.8% as AlCl_3_·6H_2_O were added to GC (3:1), the value decreases by 69.9% as compared with the basic one. It was found that MgCl_2_·6H_2_O also promoted the production of xylo-oligomers, and even better than FeCl_2_·4H_2_O; however, it only worked with GL (3:1). In general, the three inorganic metal ions could accelerate the hydrolysis of xylan fragments (the capacity order: Al^3+^ > Fe^2+^ > Mg^2+^) and there is a trade-off between xylan hydrolysis and XOSs (DP < 6) accumulation. To understand the effect of metal inorganic salts on the xylo-oligomers production, the shorter time treatments were conducted individually in GL (3:1)-based solvent systems (Fig. 2). Apparently, the xylo-oligomers yields increased when shortening the duration from 20 to 10 min for all the cases; however, the basic GL (3:1) pretreatment showed a sharp decrease of the total xylo-oligomers yields from 59.3% to 11.4%. The total xylo-oligomers yields of 10 min treatment follow the order: FeCl_2_ > AlCl_3_ > MgCl_2_, while the degree of the increment in total xylo-oligomers yield follows the trend of AlCl_3_ > FeCl_2_ > MgCl_2_. It seems that AlCl_3_ has the strongest power of accelerating the xylan hydrolysis and longer duration (20 min) led to the over-degradation of the xylo-oligomers, followed by FeCl_2_, and then MgCl_2_. This would be a great explanation for the lowest total xylo-oligomers yield for the AlCl_3_ incorporated cases. According to the work of Kang et al. [36], the capability of metal salts to accept electrons and the ionic radii of metal affect the stability of the carbohydrate complexes. The effect of the inorganic salts on the degradation of hemicellulose was supposed to follow the order: transition metal chlorides (Fe^2+^) > alkaline earth metal chlorides (Mg^2+^). What’s more, the pH of the solution also indicates the metal ions capability of promoting the xylan hydrolysis. Al^3+^ apparently formed a stronger acidic solution than other cations due to its pKa value of 4.85, followed by Fe^2+^ (9.49) and then Mg^2+^ (11.4) [37]. Therefore, FeCl_2_ possesses the moderate ability among the three metal ions to accelerate the xylan hydrolysis which is benefit for xylo-oligomers accumulation. Clearly, the results demonstrate that metal ions may synergistically work with the DES to promote the xylan hydrolysis, and the improvement varied with the types of ions. Besides, a proper treating severity which could be achieved by adding metal inorganic salts or tuning treatment time is critical for accumulation of xylo-oligomers and functional XOSs (DP < 6).

### Kinetic of xylan hydrolysis and xylo-oligomers accumulation

**Fig. 4:**
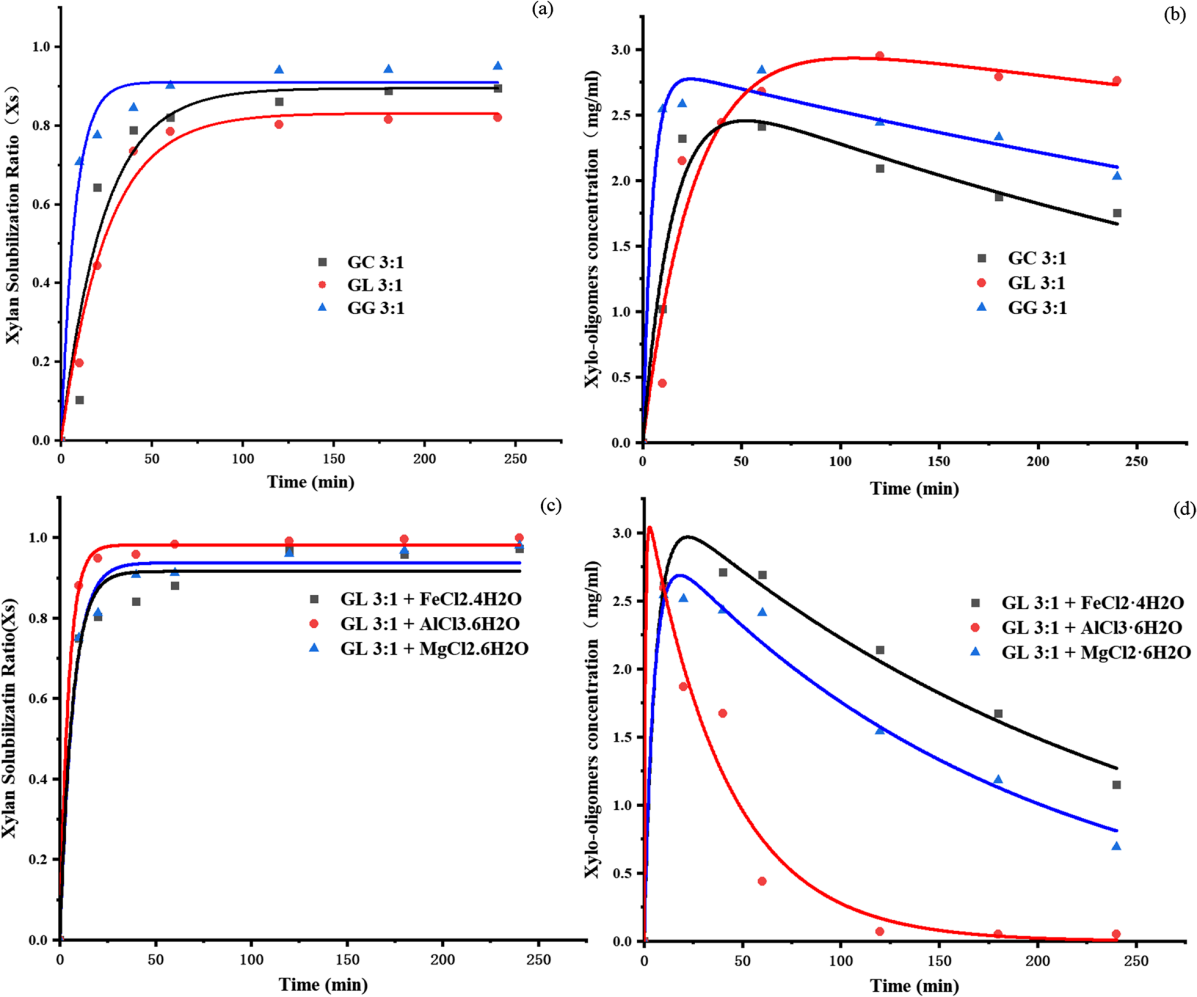
Kinetic of xylan solubilization ratio and XOSs accumulation in treatment hydrolysate. **a**–**b** The basic DES systems; **c**–**d** the metal inorganic salts incorporated DES systems. The xylan solubility and the total xylo-oligomers concentrations were present as the mean values and the data were fitted by Origin 95

To deeply explore the mechanism of xylo-oligomers formation, the kinetic study on xylan hydrolysis and xylo-oligomers production during the DES treating processes was conducted. According to Eqs. (1), (2) [16], to accurately fit the parameters *H*_d_, *k*_1_ and *k*_2_, it is necessary to determine the relationship between the X_S_ or C_xos_ values and the reaction time, and the fittings were succeed with the regression coefficient (*R*^2^) ranged from 0.91 to 0.99 (Table 2). The fitting curves of xylan solubilization and xylo-oligomers accumulation during the basic DES treating processes are shown in Fig. 4a, b, respectively. Obviously, the rate of xylan solubilization follows the trend of GG (3:1) > GC (3: 1) > GL (3: 1). For example, the Xs values of GG (3:1), GC (3:1), and GL (3:1) associated cases at 4 h were 0.9502, 0.8941, and 0.8211, respectively. Moreover, the H_d_ values (Table 2) for these systems were 0.91, 0.89, and 0.83, respectively, and these data also well reflected the xylan dissolving ability of the solvents. Hence, it was obvious that GG (3:1) is the best one for dissolving xylan. It is worth noting that the optimal solvent for xylo-oligomers accumulation is appeared to be GL (3: 1), the GG (3:1) comes the next, and the worst one is GC (3:1) (Fig. 4b). The accumulation mechanism of the total xylo-oligomers can be further understood by the other data in Table 2. The value k_1_ (reflecting the formation speed of xylo-oligomers) for the three solvents follows the order of GG (3:1) > GC (3: 1) > GL (3: 1), which indicates that the highest conversion speed of xylan to xylo-oligomers was occurred in GG (3:1) as well. However, the accumulation of xylo-oligomers not only depend on the producing speed of xylo-oligomers (value *k*_1_), but the degradation of xylo-oligomers to xylose (value *k*_2_) also needs to be considered. It can be seen in Table 2 that the values of *k*_2_ follow the order: GC (3:1) > GG (3:1) > GL (3:1). The smaller the value of *k*_2_, the slower the conversion speed of xylo-oligomers to xylose, thus contributing to more accumulation of xylo-oligomers. Therefore, according to the data showed in Table 2 and Fig. 4b, it can be concluded that the best solvent for xylo-oligomers accumulation is GL (3:1). Others’ reports pointed out that the xylo-oligomers existed only for a very short time during the treatment of sugarcane bagasse by sulfuric acid [16]. In this work, it is clear that the degradation speed of xylo-oligomers is lower as compared to its formation rate (from xylan to xylo-oligomers), and the yield of the total xylo-oligomers reached 74.5% after treating by GL (3:1) for 2 h. Hence, GL (3:1) is a promising solvent for xylo-oligomers production and worth further investigation by adding metal inorganic salts.

**Table 2:** Potential degree of hydrolysis and rate constant of xylan hydrolysis

DES	*H*_d_	*k*_1_	*k*_2_	*R*^2^
GC(3:1)	0.89	4.33 × 10^–2^	2.24 × 10^–3^	0.92
GL(3:1)	0.83	4.04 × 10^–2^	6.70 × 10^–4^	0.91
GG(3:1)	0.91	0.13	1.31 × 10^–3^	0.97
GL(3:1) + FeCl_2_·4H_2_O	0.92	0.15	4.00 × 10^–3^	0.99
GL(3:1) + AlCl_3_·6H_2_O	0.98	0.22	2.48 × 10^–2^	0.94
GL(3:1) + MgCl_2_·6H_2_O	0.93	0.14	5.52 × 10^–3^	0.97

The xylan hydrolysis and xylo-oligomers accumulation in the metal incorporated GL (3:1) systems are shown in Fig. 4c, d. The xylan solubilization rate in these solvents follows the order of AlCl_3_ > MgCl_2_ > FeCl_2_; however, the xylo-oligomers accumulation efficiency shows the opposite trend of FeCl_2_ > MgCl_2_ > AlCl_3._ It is obvious that FeCl_2_ is the most suitable solvent to produce considerable amounts of xylo-oligomers. As we can see in Table 2, the *H*_d_ values generated from the fitting model rang from 0.92 to 0.98, which significantly increased as compared with those of basic DES systems. In particular, the value reached 0.98 when AlCl_3_ was present, which reveals that Al^3+^ could effectively boost xylan hydrolysis. Interestingly, the addition of metal inorganic salts made a huge impact on k_1_, especially for AlCl_3_, which caused the value increased by approximately five times. This means that the addition of metal ions can greatly accelerate the conversion of xylan to xylo-oligomers in a very short time. However, in the meanwhile, the presence of them can also promote the conversion of xylo-oligomers into xylose. Besides, the *k*_2_ value increased by 37 times when AlCl_3_ was presented. Clearly, the degradation of xylo-oligomers was faster than their formation on this condition. Consequently, the accumulation of xylo-oligomers is a kind of trade-off, and it is better to keep fast xylan hydrolysis but simultaneously avoid the rapid over-degradation of xylo-oligomers.

## Conclusions

Glycolic acid-based DES were selected to treat corncob for obtaining xylo-oligomers and fermentable sugars. DES could remove large amounts of xylan from corncob at 120 °C for 20 min, achieving more than 60% yield of xylo-oligomers, and over 80% of polysaccharides enzymatic digestion. The presence of metal ions could effectively promote the hydrolysis of xylan and led to high selective removal of xylan. Kinetic results reveal that the metal ions can synergistically work with DES for promoting the hydrolysis of xylan. GL (3:1) was proved to be the best solvent for xylo-oligomers production, and the presence of Fe^2+^ was benefit for accumulation of xylo-oligomers. The accumulation of xylo-oligomers is a kind of trade-off, and keeping fast xylan hydrolysis and simultaneously avoiding the over-degradation of xylo-oligomers are necessary for an efficient xylo-oligomers production.

## Materials and methods

### Materials

Compound cellulase was purchased from Novozymes (China) and used as received. Corncob acquired from Guangdong, China was ground to a particle size of 65 mesh by a grinder. Then, the ground raw corncob was air-dried (water content was 6.0 wt%) before use. Choline chloride (ChCl, 98%) were purchased from Shanghai Macklin Biochemical Co., Ltd. (Macklin, Shanghai, China). Glycolic acid (98%), lactic acid (85%), guanidine hydrochloride (99%), ferrous chloride tetrahydrate (98%), aluminum chloride hexahydrate (99.99%), magnesium chloride hexahydrate (99.9%), glucose (99%), xylose (99%), and furfural (99%) were purchased from Aladdin Biochemical Technology Co., Ltd. (Aladdin, Shanghai, China). Xylobiose (98%) and Xylotriose (98%) were purchased from Shanghai Yuanye Bio-Technology Co., Ltd. (Yuanye, Shanghai, China). Xylotetraose (99%) and Xylopentaose (99%) were purchased from Shanghai ZZBIO Co., Ltd. (ZZBIO, Shanghai, China). Other chemicals were of the highest purity commercially available.

### DES preparation

Glycolic acid (G), choline chloride (C), lactic acid (L), guanidine hydrochloride (G), ferrous chloride tetrahydrate, aluminum chloride hexahydrate, and magnesium chloride hexahydrate were dried under vacuum at 80 °C for 5 h before use. The DES were prepared by mixing glycolic acid with choline chloride (GC), lactic acid (GL), and guanidine hydrochloride (GG) in a molar ratio of 1:1or 3:1. For the metal inorganic salts contained system, 5% (w/w) ferrous chloride tetrahydrate and aluminum chloride hexahydrate were added to GC (3:1), GL (3:1), and GG (3:1), respectively. Magnesium chloride hexahydrate could only dissolve in GL (3:1). The mixture was heated and stirred at a certain temperature in a closed flask until a homogenous colorless solution was formed. The prepared DES were then stored in a vacuum drying oven before use.

### Treatment of corncob with DES

Briefly, corncob samples were mixed with DES with a biomass loading of 5 wt%, and then the mixtures were stirred and kept at 120 °C for a specific time (10 min, 20 min, 40 min, 1 h, 2 h, 3 h, or 4 h). Once the treatment was completed, the residues were thoroughly washed with anhydrous ethanol and then water, and placed in a − 20 °C refrigerator for freeze-drying. After lyophilization, the samples were placed in a sealed bag and stored in a drying oven for the subsequent use.

### Compositional analysis of the corncob samples

The cellulose, xylan, lignin, and ash contents of the corncob samples were determined according to the standard NREL analytical procedure, including acid hydrolysis, the subsequent HPLC, and gravimetric analysis [38], and these experiments were conducted in duplicate. The sugars were monitored using HPLC (Agilent 1260) equipped with a Bio-Rad Aminex HPX-87H column and a refractive index detector (Agilent 1260). The mobile phase was a 5 mM sulfuric acid aqueous solution, the flow rate was 0.5 mL/min, and the column and detector temperatures were 65 and 50 °C, respectively. The retention times for glucose and xylose were 11.4 and 12.2 min respectively.

### Enzymatic hydrolysis of the corncob samples

Enzymatic hydrolysis was implemented by mixing 20 mg of biomass and 8.3 U mL^−1^ cellulase in 7 mL of a citrate buffer (50 mmol L^−1^, pH 4.8) with stirring (120 rpm) at 50 °C. Aliquot samples (300 μL) were extraction at specified time spaces and boiled for 5 min to quench the enzymatic reaction. After being filtrated through a 0.22 μm membrane, the glucose and xylose concentrations were detected using the HPLC, as described above. All reactions were performed in duplicate, and the related data showed as mean values with standard derivations. The polysaccharide digestibility were calculated as follows:

Polysaccharide digestibility (%) = (released sugar amount)/Theoretic sugar amount in the sample used for enzymatic hydrolysis × 100.

### Fourier transform infrared spectroscopy (FT-IR) analysis

FT-IR patterns of corncob and DES to treat solids were tested from an FT-IR (Nexus Thermo Nicolet, USA). The samples for test were combined with KBr (1/10 mass ratio), evenly grinding both and to press into flakes with 7 MPa and 30 s. 32 background and scans were taken from 400 to 4000 cm^−1^.

### Analysis of the total xylo-oligomers and XOSs (DP 2–5)

To collect the total xylo-oligomers, deionized water of a certain volume was added after the reaction completed, and the mixture was centrifuged at 10,000 g for 5 min. The supernatant was collected. The xylo-oligomers measurement was conducted according to the previous report [18]. Briefly, the supernatant was obtained by centrifugation. Then, the supernatant was hydrolyzed with 4% H_2_SO_4_ at 121 °C for 1 h. The total xylo-oligomers yield was calculated based on the discrepancy between the content of xylose before and after hydrolysis. The equation was showed as follows:

$${\text{Total xylo}} - {\text{oligomers yield }}\left( \% \right) \, = \, \left( {{\text{xylose amount after acidolysis }} - {\text{ xylose amount before acidolysis}}} \right) \, \times 0.{88 }/{\text{Xylan amount in the raw corncob}} \times {1}00.$$

XOSs (DP 2–5) were analyzed based on the previous report [39]. The XOSs (DP 2–5) were monitored using HPLC (Waters 1525) equipped with an Agilent Hi-Plex Na column and a refractive index detector. The mobile phase was water, the flow rate was 0.3 mL/min, and the column and detector temperatures were 80 °C and 50 °C, respectively. The equation was expressed as follows:

$${\text{XOSs }}\left( {{\text{DP 2}} - {5}} \right){\text{ yield }}\% \, = \, \left( {{\text{X2}} + {\text{X3}} + {\text{X4}} + {\text{X5 in liquor}}} \right) \, /{\text{Xylan amount in the raw corncob}} \times {1}00.$$

### Mass balance of xylan

Furfural were analyzed based on our previous report [24]. The furfural was monitored using HPLC (Waters 2695) equipped with an Agilent C-18 column and a DAD detector at 280 nm. The mobile phase was acetonitrile/water (15/85, v/v), and the flow rate was 1 mL/min^−1^. The equation was expressed as follows:

$${\text{Conversion of Furfural }}\left( \% \right) \, = \, \left( {{\text{furfural amount in liquid}} \times {1}.{375}} \right) \, /{\text{Total xylan and arabinan amount in raw corncob}} \times {1}00$$

$${\text{Residual xylan }}\left( \% \right) \, = \, \left( {\text{xylan amount in corncob residues}} \right)/{\text{ Xylan amount in the raw corncob}} \times {1}00$$

$${\text{Xylan loss }}\left( \% \right) \, = { 1} - {\text{ Residual xylan }} - {\text{ Conversion }}\left( {{\text{xylo}} - {\text{oligomers}}} \right) \, - {\text{ Conversion }}\left( {{\text{xylose}}} \right) - {\text{ Conversion }}\left( {{\text{furfural}}} \right).$$

### Kinetic study of xylan hydrolysis and xylo-oligomers accumulation

Kinetic models used in this study were referred as the previous report [16]. Briefly, DES of six kinds, GC (3:1), GL (3:1), GG (3:1), GL (3:1)/FeCl_2_·4H_2_O, GL (3:1)/AlCl_3_·6H_2_O, and GL (3:1) /MgCl_2_·6H_2_O were kept at 120℃ for 10 min, 20 min, 40 min, 1 h, 2 h, 3 h, and 4 h to treat corncob, and the content percentage of xylan in the residue and the concentration of xylo-oligomers in supernatant at different times were determined according to the above-mentioned methods. Origin 95 was used to fit the models. Xylan solubility and xylo-oligomers concentration were fitted according to the formula listed as follows:

1 $${\text{Xs}}\,{ = }\,{\text{H}}_{{\text{d}}} \,{ - }\,{\text{exp}}\left( {-{\text{k}}_{{1}} {\text{t}}} \right)$$

2 $${\text{C}}_{{{\text{XOS}}}} \,{ = }\,\frac{{{1}{\text{.136C}}_{{0}} {\text{k}}_{{1}} {\text{H}}_{{\text{d}}} }}{{{\text{k}}_{{2}} \,{ - }\,{\text{k}}_{{1}} }}\left[ {{\text{exp}}\left( {-{\text{k}}_{{1}} {\text{t}}} \right)-{\text{exp}}\left( {-{\text{k}}_{{2}} {\text{t}}} \right)} \right],$$

where *H*_d_ is the ‘potential degree of hydrolysis’ of xylan, and 0 ≤ *H*_d_ ≤ 1; C_0_ is the initial concentration of xylan in the pseudo-homogeneous system (mg/ml); C_xos_ is the concentration of the total xylo-oligomers in the treatment liquor (mg/mL); 1.136 is the conversion coefficient of xylan into xylose.

## Data Availability

All the data are available for publication and information used from online resources has been cited properly. All data generated or analyzed during this study are included in this published article.

## Abbreviations

DES: Deep eutectic solvents
GC: Glycolic acid/choline chloride
GL: Glycolic acid/lactic acid
GG: Glycolic acid/guanidine hydrochloride
GC (3:1)/FeCl2·4H2O: Glycolic acid/choline chloride/ferrous chloride tetrahydrate
GC (3:1)/AlCl3·6H2O: Glycolic acid/lactic acid/aluminum chloride hexahydrate
GG (3:1)/MgCl2·6H2O: Glycolic acid/guanidine hydrochloride/magnesium chloride hexahydrate
